# Supplementary material for: Removing reference bias and improving indel calling in ancient DNA data analysis by mapping to a sequence variation graph
Source: Genome Biol. 2020 Sep 17;21:250. doi: 10.1186/s13059-020-02160-7 (PMC7499850; doi:10.1186/s13059-020-02160-7)
Supplement: Supplementary file 1 — Additional file 1 Figures S1-S23 and Tables S1-S7. [file 13059_2020_2160_MOESM1_ESM.pdf]

Additional file 1
Removing reference bias and improving indel calling in ancient DNA data analysis by mapping to a sequence variation graph

Rui Martiniano[1*], Erik Garrison[2,3*], Eppie R. Jones[4], Andrea Manica[4], Richard Durbin[1,2#]

1. Department of Genetics, University of Cambridge, Cambridge CB3 0DH, UK
2. Wellcome Sanger Institute, Cambridge CB10 1SA, UK
3. Genomics Institute, University of California, Santa Cruz, CA 95064, USA
4. Department of Zoology, University of Cambridge, Cambridge CB2 3EJ, UK

* Equal contribution

# Correspondence to richard.durbin@gen.cam.ac.uk

List of Figures

S1 Diagram summarizing the data processing workflow 11
S2 Comparison of the percentage of mapped reads in simulated data between vg and bwa aln 12
S3 Comparison of the percentage of mapped reads in simulated data between vg graph, vg linear reference and bwa mem 13
S4 Comparison of the percentage of mapped reads in simulated data between vg and bwa aln and modreads 14
S5 Comparison of the percentage of mapped reads in simulated data between vg and the Peyrégne method 15
S6 Comparison of the percentage of incorrectly mapped reads between bwa, vg and vg linear reference at different mapping quality filtering thresholds 16
S7 Relationship between deamination and alignment error in vg and bwa alignments . . 17
S8 Circos plots comparing the types of errors observed in all the simulated reads aligned with bwa and vg 18
S9 Comparison of spurious alignment of simulated microbial sequences of different read lengths (30-100bp) between bwa aln and bwa mem to the human reference genome and vg to the 1000GP graph 19
S10 Investigation of the effect of deamination levels in the spurious alignment of simulated microbial sequences of different read lengths (30, 50, 70 and 90bp) in bwa aln, bwa mem and vg 20
S11 X-chromosome contamination estimates 21
S12 Comparison between deamination patterns in sequence read data belonging to a high-coverage Yamnaya sample processed with vg and bwa aln and filtered with different mapping quality thresholds 22
S13 Comparison of deamination and softclipping patterns in sequences with artificially introduced C to T changes in the 1st and 2nd nucleotides of the 5'-end of sequencing reads. 23
S14 Comparison between vg and bwa in terms of alternate allele support in the Yamnaya sample 24
S15 Comparison between vg and bwa in terms of alternate allele support in the Yamnaya sample 25
S16 Comparison of indel calling between the Yamnaya sample processed with vg graph and bwa aln. 26
S17 D-statistic test of reference bias in aDNA based on real data, using Chimp as an outgroup and including transversion SNPs only 27
S18 D-statistic test of reference bias comparing bwa aln and vg, alt allele 28
S19 D-statistic test of reference bias in aDNA on simulated data, using Chimp as an outgroup and including transversion SNPs only 29
S20 D-statistic test of reference bias comparing bwa-modread and vg 30
S21 Principal Component Analysis estimated with present-day West Eurasians from the Human Origins dataset with ancient samples aligned with either vg graph or bwa aln 31
S22 PCA of a subset of the Human Origins to examine the effect of read length in PC coordinates 32

S23 Principal Component Analysis restricted to indels identified in Yamnaya processed with bwa and vg . . . 33

List of Tables

S1 Alternate allele fraction obtained after alignment of simulated data with vg, bwa aln and bwa mem, using different parameters and mapping quality filter thresholds. . . . 4
S2 Comparison of the mean percentage of incorrectly mapped reads between vg alignment to the 1000GP graph, vg, bwa aln and bwa mem alignment to the linear reference at different mapping quality thresholds and alignment parameters. Error rates for the workflow which requires alignment to two reference genomes are also shown ('altref_genome'). . . . 5
S3 Comparison of the percentage of incorrectly aligned microbial reads of different lengths (30 to 100 bp) to the human reference genome between bwa aln, bwa mem and vg graph. . . . 6
S4 Detailed description of the ancient DNA data processed in the present study. . . . 7
S5 Number of mapped reads, genomic coverage and endogenous content after aligning sequencing reads with vg to the 1000 Genomes variation graph. . . . 8
S6 Number of mapped reads, genomic coverage and endogenous content after aligning sequencing reads with bwa aln to the human reference genome. . . . 9
S7 Comparison of run times, memory and storage between bwa and vg for the indexing of the linear human reference genome and the 1000GP graph, respectively. We also compare read alignment and bam generation with vg, bwa aln and bwa mem, using an uncompressed FASTQ file containing 10 million simulated 50 bp reads with various levels of deamination. . . . 10

Table S1: Alternate allele fraction obtained after alignment of simulated data with vg, bwa aln and bwa mem, using different parameters and mapping quality filter thresholds.

aligner	mean alternate allele fraction and 95% CI
vg graph q30	0.50001 [0.50000,0.50003]
vg graph q50	0.49988 [0.49984,0.49991]
vg graph q60	0.49933 [0.49924,0.49941]
vg linear q50	0.49555 [0.49510,0.49600]
bwa mem q50	0.48987 [0.48902,0.49073]
bwa aln -n 0.02 q25	0.49705 [0.49661,0.49750]
bwa aln -n 0.02 q30	0.48267 [0.48095,0.48438]
bwa aln -n 0.02 modreads q25	0.50015 [0.50014,0.50017]
bwa aln -n 0.02 modreads q30	0.50074 [0.50071,0.50077]
bwa aln -n 0.02 altref genome q25	0.50009 [0.50009,0.50009]
bwa aln -n 0.02 altref genome q30	0.49997 [0.49996,0.49999]
bwa aln -n 0.01 -o2 q25	0.49936 [0.49927,0.49945]
bwa aln -n 0.01 -o2 q30	0.49702 [0.49657,0.49747]
bwa aln n 0.01 -o2 modreads q25	0.50002 [0.50002,0.50003]
bwa aln n 0.01 -o2 modreads q30	0.50016 [0.50014,0.50017]
bwa aln n 0.01 -o2 altref genome q25	0.50011 [0.50011,0.50011]
bwa aln n 0.01 -o2 altref genome q30	0.50009 [0.50009,0.50009]

Table S2: Comparison of the mean percentage of incorrectly mapped reads between vg alignment to the 1000GP graph, vg, bwa aln and bwa mem alignment to the linear reference at different mapping quality thresholds and alignment parameters. Error rates for the workflow which requires alignment to two reference genomes are also shown ('altref_genome').

				**mean error REF (%)**			
**mapping quality threshold**	**vg graph**	**vg linear**	**bwa mem**	**bwa aln -n0.02**	**bwa aln -n0.01 -o 2**	**bwa aln -n0.02 altref genome**	**bwa aln -n0.01 -o 2 altref genome**
**q>0**	0.00714	0.00694	0.00315	0.00168	0.00168	NA	NA
**q>=25**	0.00018	0.00019	0.00024	0.00005	0.00002	0.00108	0.00062
**q>=30**	0.00015	0.00017	0.00016	0.00002	0.00001	0.00053	0.00054
**q>=50**	0.00012	0.00013	0.00007	NA	NA	NA	NA
**q>=60**	0.00011	0.00012	0.00004	NA	NA	NA	NA
				**mean error ALT (%)**			
	**vg graph**	**vg linear**	**bwa mem**	**bwa aln -n0.02**	**bwa aln -n0.01 -o 2**	**bwa aln -n0.02 altref genome**	**bwa aln -n0.01 -o 2 altref genome**
**q>0**	0.01231	0.06035	0.02824	0.00980	0.00971	NA	NA
**q>=25**	0.00044	0.00191	0.00309	0.00054	0.00025	0.00051	0.00024
**q>=30**	0.00037	0.00162	0.00205	0.00024	0.00020	0.00021	0.00020
**q>=50**	0.00025	0.00110	0.00087	NA	NA	NA	NA
**q>=60**	0.00021	0.00097	0.00056	NA	NA	NA	NA
				**mean error All (%)**			
	**vg graph**	**vg linear**	**bwa mem**	**bwa aln -n0.02**	**bwa aln -n0.01 -o 2**	**bwa aln -n0.02 altref genome**	**bwa aln -n0.01 -o 2 altref genome**
**q>0**	0.00972	0.03360	0.01563	0.00571	0.00569	NA	NA
**q>=25**	0.00031	0.00105	0.00165	0.00030	0.00013	0.0008	0.00043
**q>=30**	0.00026	0.00089	0.00109	0.00012	0.00011	0.00037	0.00037
**q>=50**	0.00019	0.00061	0.00046	NA	NA	NA	NA
**q>=60**	0.00016	0.00054	0.00029	NA	NA	NA	NA

Table S3: Comparison of the percentage of incorrectly aligned microbial reads of different lengths (30 to 100 bp) to the human reference genome between bwa aln, bwa mem and vg graph.

**Read length**	**bwa aln -n0.01 -o2**		**bwa aln -n0.02**		**bwa mem**			**vg graph**		
	**q>=25**	**q>=30**	**q>=25**	**q>=30**	**q>=30**	**q>=50**	**q>=60**	**q>=30**	**q>=50**	**q>=60**
**30**	2.372	1.329	1.94	0.897	0.007	0.001	0.001	2.31	0.644	0.055
**35**	0.582	0.253	0.525	0.196	0.041	0.017	0.007	1.194	0.12	0.014
**40**	0.217	0.06	0.207	0.05	0.063	0.044	0.034	0.66	0.054	0.01
**45**	0.219	0.076	0.085	0.022	0.146	0.094	0.061	0.409	0.017	0.003
**50**	0.146	0.06	0.077	0.027	0.157	0.087	0.069	0.328	0.034	0.008
**55**	0.115	0.05	0.115	0.05	0.212	0.153	0.102	0.268	0.045	0.023
**60**	0.071	0.02	0.071	0.02	0.218	0.146	0.122	0.244	0.057	0.031
**65**	0.08	0.022	0.022	0.004	0.246	0.174	0.12	0.239	0.077	0.043
**70**	0.036	0.009	0.009	0	0.326	0.234	0.188	0.256	0.111	0.074
**75**	0.014	0.002	0.003	0	0.33	0.217	0.168	0.26	0.128	0.072
**80**	0.004	0.001	0.004	0.001	0.417	0.286	0.209	0.281	0.153	0.099
**85**	0	0	0	0	0.442	0.299	0.209	0.291	0.165	0.118
**90**	0.002	0	0	0	0.477	0.316	0.218	0.326	0.196	0.154
**95**	0.001	0.001	0.001	0.001	0.539	0.378	0.269	0.343	0.21	0.153
**100**	0	0	0	0	0.54	0.374	0.246	0.353	0.248	0.196

Table S4: Detailed description of the ancient DNA data processed in the present study.

Set	Publication	SampleID	Type	Treatment	Reported coverage	Geographical Region
1	Damgaard et al., 2018	Yamnaya	Shotgun	untreated	13.6	Kazakhstan
1		Botai	Shotgun	untreated	25.2	Kazakhstan
2	Martiniano et al., 2016	1489	Shotgun	untreated	0.6	United Kingdom
2		3DT16	Shotgun	untreated	0.7	United Kingdom
2		3DT26	Shotgun	untreated	1.1	United Kingdom
2		6DT18	Shotgun	untreated	1.1	United Kingdom
2		6DT21	Shotgun	untreated	1.2	United Kingdom
2		6DT22	Shotgun	untreated	1.1	United Kingdom
2		6DT23	Shotgun	untreated	0.7	United Kingdom
2		6DT3	Shotgun	untreated	1.7	United Kingdom
2		NO3423	Shotgun	untreated	1.1	United Kingdom
3	Schiffels et al., 2016	12880A	Shotgun	USER	1.3	United Kingdom
3		12881A	Shotgun	USER	4.4	United Kingdom
3		12883A	Shotgun	USER	3.8	United Kingdom
3		12884A	Shotgun	USER	11.8	United Kingdom
3		12885A	Shotgun	USER	0.9	United Kingdom
3		15558A	Shotgun	UDGhalf	3.8	United Kingdom
3		15569A	Shotgun	UDGhalf	2.7	United Kingdom
3		15570A	Shotgun	UDGhalf	8.2	United Kingdom
3		15577A	Shotgun	UDGhalf	6.3	United Kingdom
3		15579A	Shotgun	UDGhalf	1.4	United Kingdom
4	Posth et al., 2018	CP21half	Capture	UDGhalf	2.5	Brazil
4		CP22half	Capture	UDGhalf	2.0	Brazil
4		CP29half	Capture	UDGhalf	5.3	Peru
4		CP8half	Capture	UDGhalf	7.7	Peru
4		CUN008	Capture	UDGhalf	2.2	Peru
4		LAP003	Capture	UDGhalf	0.3	Brazil
4		LAP004	Capture	UDGhalf	1.2	Brazil
4		LAP005	Capture	UDGhalf	2.1	Brazil
4		LAP006	Capture	UDGhalf	0.9	Brazil
4		LAP007	Capture	UDGhalf	0.7	Brazil
4		LAR001	Capture	UDGhalf	0.6	Brazil
4		LAR002	Capture	UDGhalf	0.4	Brazil
4		MOS001	Capture	UDGhalf	0.4	Brazil

Table S5: Number of mapped reads, genomic coverage and endogenous content after aligning sequencing reads with vg to the 1000 Genomes variation graph.

sampleID	number of reads	vg graph								
		aligned_mapq30_rmdups	endogenous_q30	coverage_q30	aligned_mapq50_rmdups	endogenous_q50	coverage_q50	aligned_mapq60_rmdups	endogenous_q60	coverage_q60
Botai	1865598223	421934878	22.617	11.8103X	395242184	21.186	11.3872X	383123781	20.536	11.1798X
Yamnaya	2663552613	804045151	30.187	19.3594X	758072353	28.461	18.5996X	734846662	27.589	18.211X
1489	81132543	22320156	27.511	0.5485X	21344354	26.308	0.5296X	20859215	25.710	0.5199X
3DT16	62769508	28831835	45.933	0.6661X	27452525	43.735	0.6394X	26767136	42.644	0.6259X
3DT26	184353713	48564256	26.343	1.1371X	46335474	25.134	1.0956X	45249933	24.545	1.0745X
6DT18	97642161	40381525	41.357	1.0543X	38905880	39.845	1.0208X	38182006	39.104	1.0041X
6DT21	91458699	47424630	51.854	1.152X	45480536	49.728	1.111X	44513919	48.671	1.0904X
6DT22	114753712	46199468	40.260	1.1107X	44218645	38.534	1.0708X	43242540	37.683	1.0508X
6DT23	116564616	25504242	21.880	0.6459X	24434218	20.962	0.6237X	23927824	20.528	0.6126X
6DT3	111539058	68660843	61.558	1.6627X	65793129	58.987	1.6028X	64354962	57.697	1.5725X
NO3423	89138342	43629793	48.946	1.0175X	41665842	46.743	0.9792X	40673417	45.630	0.9596X
12880A	829809746	56290616	6.784	0.9581X	52677856	6.348	0.9108X	51310381	6.183	0.8903X
12881A	794714619	172620548	21.721	2.7813X	161674460	20.344	2.6227X	157181011	19.778	2.553X
12883A	773030552	153957911	19.916	2.591X	145022936	18.760	2.4591X	141268741	18.275	2.3996X
12884A	1321644764	432117158	32.695	7.5554X	410492176	31.059	7.2076X	400887929	30.333	7.0476X
12885A	497373675	38872627	7.816	0.6481X	36119828	7.262	0.6131X	35100206	7.057	0.5982X
15558A	1054098249	152745125	14.491	2.8399X	144221781	13.682	2.6959X	140341229	13.314	2.6284X
15569A	237401000	93134209	39.231	1.6552X	87821202	36.993	1.5664X	85337285	35.946	1.5244X
15570A	823653962	299364605	36.346	5.2394X	284333699	34.521	4.9962X	277262836	33.663	4.8806X
15577A	683617585	232774127	34.050	4.3753X	221020584	32.331	4.1814X	215405385	31.510	4.0866X
15579A	327301910	55597145	16.987	0.7743X	51828213	15.835	0.727X	49962384	15.265	0.7029X
CP21half	29829944	6125209	20.534	0.1219X	5540251	18.573	0.112X	5279948	17.700	0.1073X
CP22half	24599607	5372420	21.839	0.0997X	4806622	19.539	0.0904X	4559164	18.533	0.086X
CP29half	26634662	13403728	50.324	0.2736X	12541204	47.086	0.2578X	12141458	45.585	0.2502X
CP8half	33014602	18898577	57.243	0.3924X	17536032	53.116	0.3669X	16915888	51.238	0.3548X
CUN008	24348976	6701266	27.522	0.1367X	5971646	24.525	0.1231X	5636410	23.148	0.1165X
LAP003	34760577	1112049	3.199	0.0174X	883129	2.541	0.0148X	805044	2.316	0.0137X
LAP004	79550494	4897788	6.157	0.0825X	4084170	5.134	0.0711X	3767481	4.736	0.0661X
LAP005	101557239	9350735	9.207	0.1696X	7954168	7.832	0.1479X	7374831	7.262	0.1379X
LAP006	62623053	4189609	6.690	0.0644X	3596808	5.744	0.0567X	3361499	5.368	0.0533X
LAP007	51237871	3322559	6.485	0.0512X	2744928	5.357	0.0437X	2527904	4.934	0.0406X
LAR001	12623337	1853770	14.685	0.0289X	1630946	12.920	0.0256X	1538141	12.185	0.0241X
LAR002	10812910	1249986	11.560	0.0197X	1076414	9.955	0.0172X	1008331	9.325	0.0161X
MOS001	17259402	1164601	6.748	0.0171X	996820	5.776	0.0148X	933975	5.411	0.0139X

Table S6: Number of mapped reads, genomic coverage and endogenous content after aligning sequencing reads with bwa aln to the human reference genome.

sampleID	number of reads	bwa -n 0.02						bwa -n 0.01 -o 2					
		aligned_mapq25_rmdups	endogenous_q25	coverage_q25	aligned_mapq30_rmdups	endogenous_q30	coverage_q30	aligned_mapq25_rmdups	endogenous_q25	coverage_q25	aligned_mapq30_rmdups	endogenous_q30	coverage_q30
Botai	1865598223	416223409	22.310	11.3479X	410152233	21.985	11.2361X	415164292	22.254	11.274X	410749213	22.017	11.1976X
Yamnaya	2663552613	811584947	30.470	19.1319X	801807300	30.103	18.952X	809244367	30.382	19.0093X	802441463	30.127	18.8915X
1489	81132543	22535412	27.776	0.5518X	22138624	27.287	0.5428X	22514582	27.750	0.5505X	22256397	27.432	0.545X
3DT16	62769508	28595404	45.556	0.6614X	28220816	44.959	0.6531X	28553146	45.489	0.6596X	28323320	45.123	0.6547X
3DT26	184353713	47957571	26.014	1.1258X	47211710	25.609	1.1091X	47913845	25.990	1.1234X	47443675	25.735	1.1133X
6DT18	97642161	40243643	41.215	1.0492X	39968525	40.934	1.0425X	40102223	41.071	1.0446X	39952714	40.917	1.0412X
6DT21	91458699	47102981	51.502	1.143X	46659138	51.017	1.1326X	46986468	51.375	1.1389X	46724635	51.088	1.1331X
6DT22	114753712	45849456	39.955	1.1022X	45384992	39.550	1.0918X	45753752	39.871	1.0987X	45474863	39.628	1.0927X
6DT23	116564616	25166305	21.590	0.6388X	24866360	21.333	0.6315X	25101512	21.534	0.6365X	24925776	21.384	0.6324X
6DT3	111539058	68144820	61.095	1.6488X	67425973	60.451	1.6322X	67992471	60.958	1.6433X	67564411	60.575	1.6339X
NO3423	89138342	44498077	49.920	1.0355X	43903918	49.254	1.0225X	44405123	49.816	1.0318X	44037034	49.403	1.0241X
12880A	829809746	58594810	7.061	1.0066X	58508458	7.051	1.0052X	58588180	7.060	1.0063X	58522112	7.052	1.0052X
12881A	794714619	185542021	23.347	2.9876X	185119809	23.294	2.9811X	185574034	23.351	2.9877X	185217661	23.306	2.9824X
12883A	773030552	162441204	21.014	2.7352X	162132983	20.974	2.7303X	162436150	21.013	2.7346X	162187543	20.981	2.7308X
12884A	1321644764	451142252	34.135	7.8728X	450344000	34.075	7.8596X	451081278	34.130	7.87X	450442538	34.082	7.8598X
12885A	497373675	40245318	8.092	0.6794X	40172448	8.077	0.6782X	40246069	8.092	0.6792X	40188524	8.080	0.6784X
15558A	1054098249	159809718	15.161	2.9646X	159401847	15.122	2.9575X	159759312	15.156	2.9627X	159448052	15.126	2.9574X
15569A	237401000	97393061	41.025	1.7286X	97202437	40.944	1.7253X	97378920	41.019	1.728X	97239275	40.960	1.7257X
15570A	823653962	310627818	37.713	5.4285X	310093622	37.649	5.4196X	310588471	37.709	5.4267X	310179713	37.659	5.4201X
15577A	683617585	242272982	35.440	4.5412X	241843836	35.377	4.5337X	242195248	35.428	4.5384X	241880760	35.382	4.5331X
15579A	327301910	57857227	17.677	0.8094X	57716758	17.634	0.8074X	57855021	17.676	0.8093X	57749295	17.644	0.8079X
CP21half	29829944	6409908	21.488	0.1286X	6377413	21.379	0.1279X	6413170	21.499	0.1286X	6394889	21.438	0.1283X
CP22half	24599607	5695703	23.154	0.1064X	5661549	23.015	0.1057X	5698772	23.166	0.1064X	5678398	23.083	0.106X
CP29half	26634662	13862670	52.047	0.2831X	13799520	51.810	0.2818X	13870225	52.076	0.2833X	13833883	51.939	0.2826X
CP8half	33014602	19322936	58.528	0.4029X	19145655	57.991	0.3992X	19356554	58.630	0.4036X	19234195	58.260	0.4012X
CUN008	24348976	7232279	29.703	0.1466X	7198507	29.564	0.1459X	7231444	29.699	0.1466X	7205168	29.591	0.146X
LAP003	34760577	1096188	3.154	0.0184X	1086514	3.126	0.0183X	1098299	3.160	0.0185X	1090573	3.137	0.0184X
LAP004	79550494	5184747	6.518	0.0898X	5141548	6.463	0.0891X	5191313	6.526	0.0899X	5160397	6.487	0.0894X
LAP005	101557239	10078860	9.924	0.1854X	10006121	9.853	0.1841X	10087457	9.933	0.1856X	10037619	9.884	0.1847X
LAP006	62623053	4346951	6.941	0.0687X	4308189	6.880	0.068X	4351282	6.948	0.0687X	4323436	6.904	0.0683X
LAP007	51237871	3425557	6.686	0.0547X	3400460	6.637	0.0543X	3429966	6.694	0.0548X	3411388	6.658	0.0545X
LAR001	12623337	2014020	15.955	0.0316X	2001981	15.859	0.0314X	2014625	15.960	0.0316X	2005651	15.888	0.0315X
LAR002	10812910	1383937	12.799	0.0221X	1376256	12.728	0.022X	1384288	12.802	0.0221X	1378604	12.750	0.022X
MOS001	17259402	1263689	7.322	0.019X	1255509	7.274	0.0188X	1264502	7.326	0.019X	1258051	7.289	0.0189X

Table S7: Comparison of run times, memory and storage between bwa and vg for the indexing of the linear human reference genome and the 1000GP graph, respectively. We also compare read alignment and bam generation with vg, bwa aln and bwa mem, using an uncompressed FASTQ file containing 10 million simulated 50 bp reads with various levels of deamination.

**Process**	**Command**	**CPU time**	**Max Memory (Gb)**	**Storage (Gb)**
Indexing	bwa index hsGR37.fa	02:11:04	4.5	5.2
	vg index 1000GP_AF0.001	26:40:14	121.1	38.1
Read alignment and bam file generation	vg map -w 1024 -k15 –surject-to bam	06:17:56	37.3	-
	bwa mem + samtools view -Sb	00:13:04	5.4	-
	bwa aln -t1 -l1024 -q15 -n0.01 -o 2 + bwa samse + samtools view -Sb	01:16:16	4.6	-
	bwa aln -t1 -l1024 -q15 -n0.01 + bwa samse + samtools view -Sb	01:22:43	3.9	-
	bwa aln -t1 -l1024 -q15 -n0.02 + bwa samse + samtools view -Sb	00:48:48	4.6	-

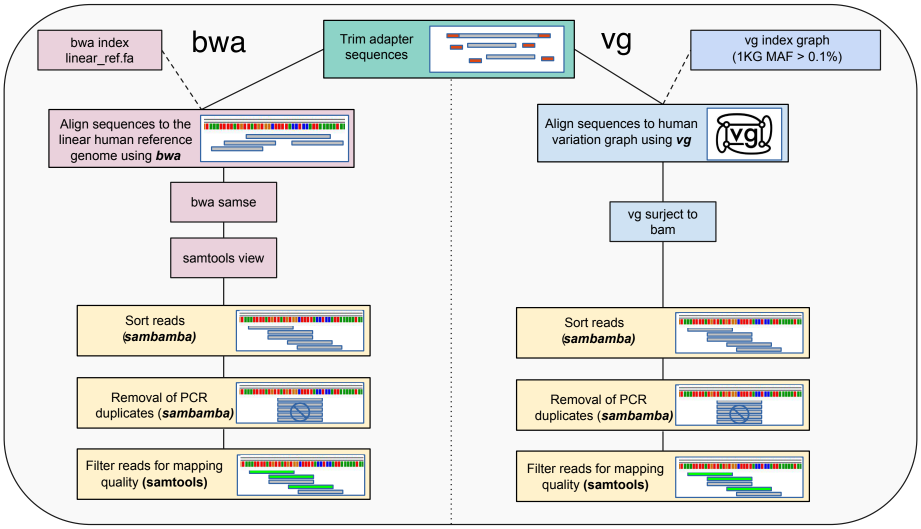

Fig. S1: Diagram summarizing the data processing workflow.

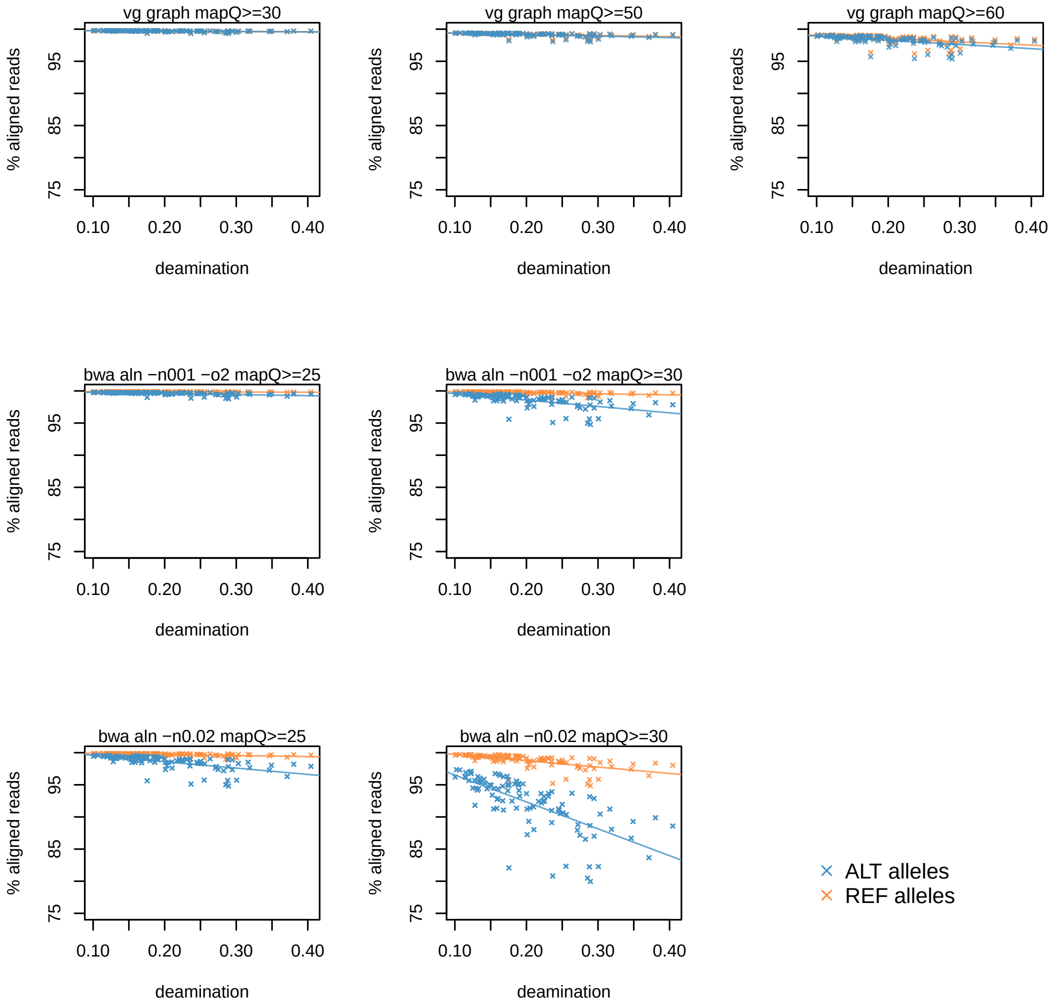

Fig. S2: Comparison of the percentage of mapped reads in simulated data between vg graph and bwa aln (-n 0.02 and -n 0.01 -o 2) filtered with different mapping quality thresholds.

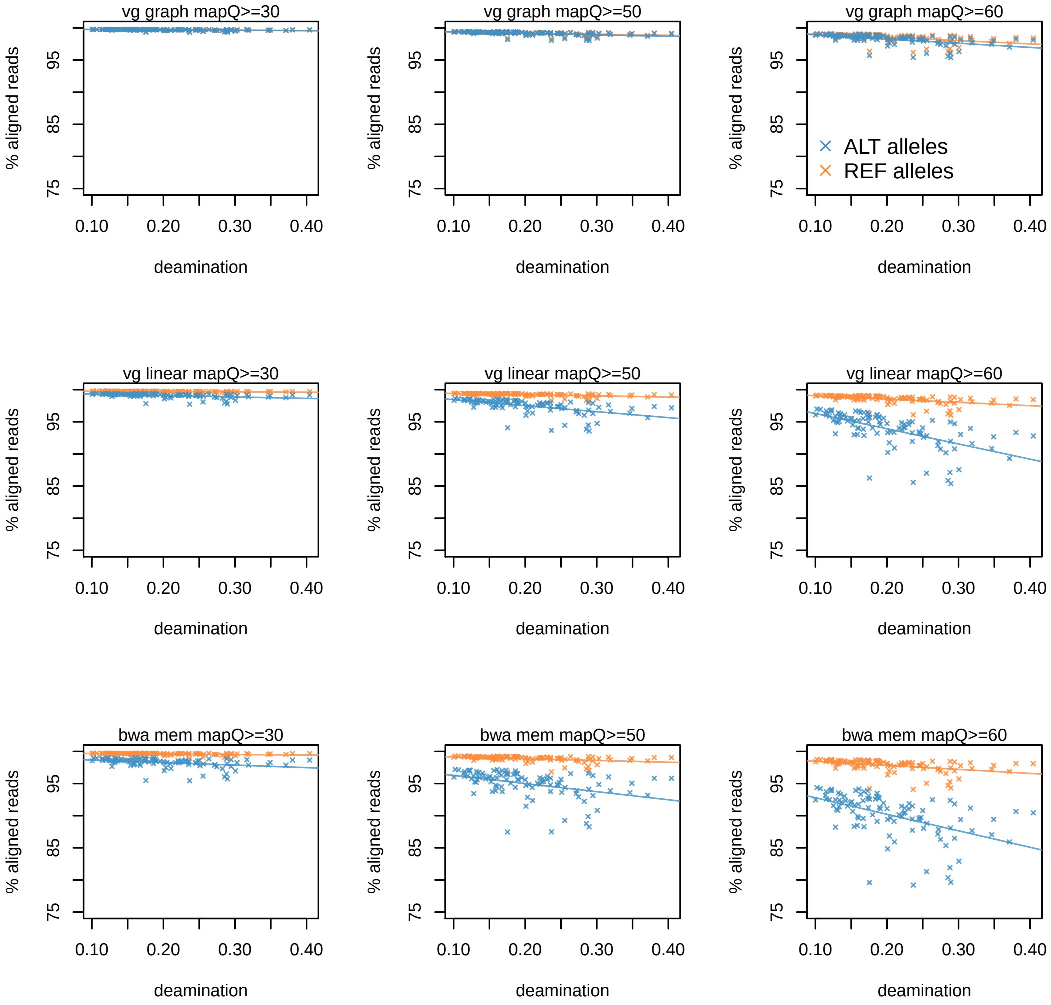

Fig. S3: Comparison of the percentage of mapped reads in simulated data between vg graph, vg linear reference and bwa mem filtered with different mapping quality thresholds.

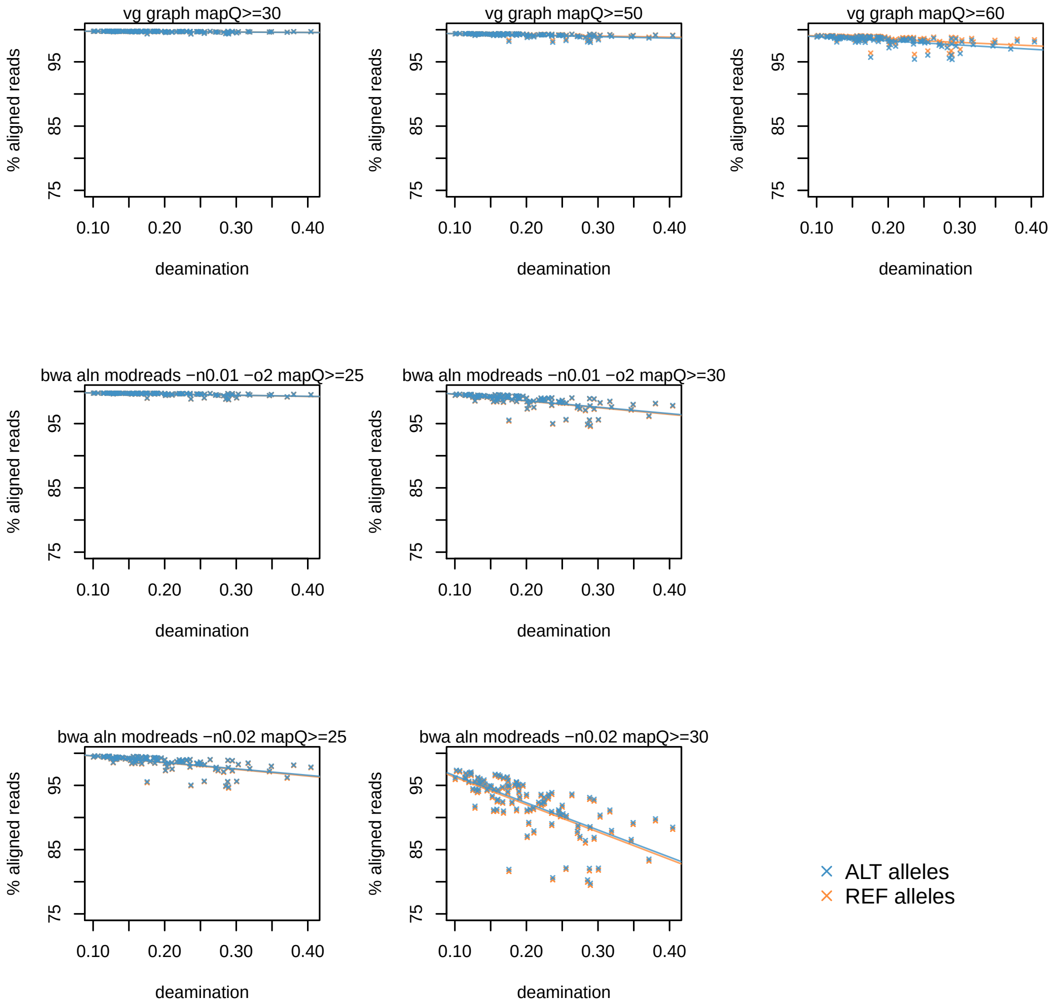

Fig. S4: Comparison of the percentage of mapped reads in simulated data between vg graph and bwa aln (-n 0.02 and -n 0.01 -o 2) after processing with the Günther & Nettelblad method which modifies reads to remove reference bias.

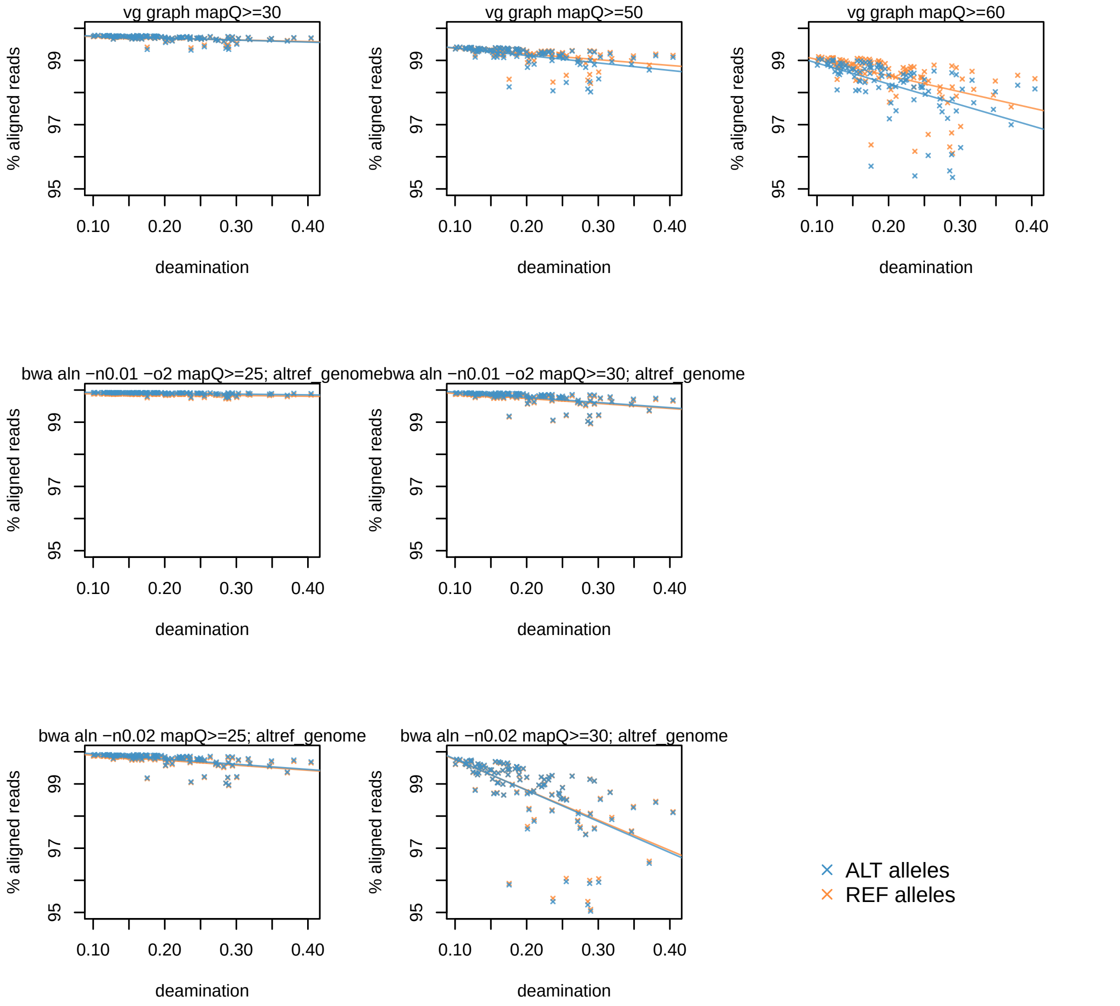

Fig. S5: Comparison of the percentage of mapped reads in simulated data between vg and bwa aln (-n 0.02 and -n 0.01 -o 2) and post processing with the Peyrégne workflow, in which reads are mapped to two versions of the reference genome (one with the alternate and the other with the reference allele at Human Origins SNPs; 'altref_genome') in order to remove reference bias.

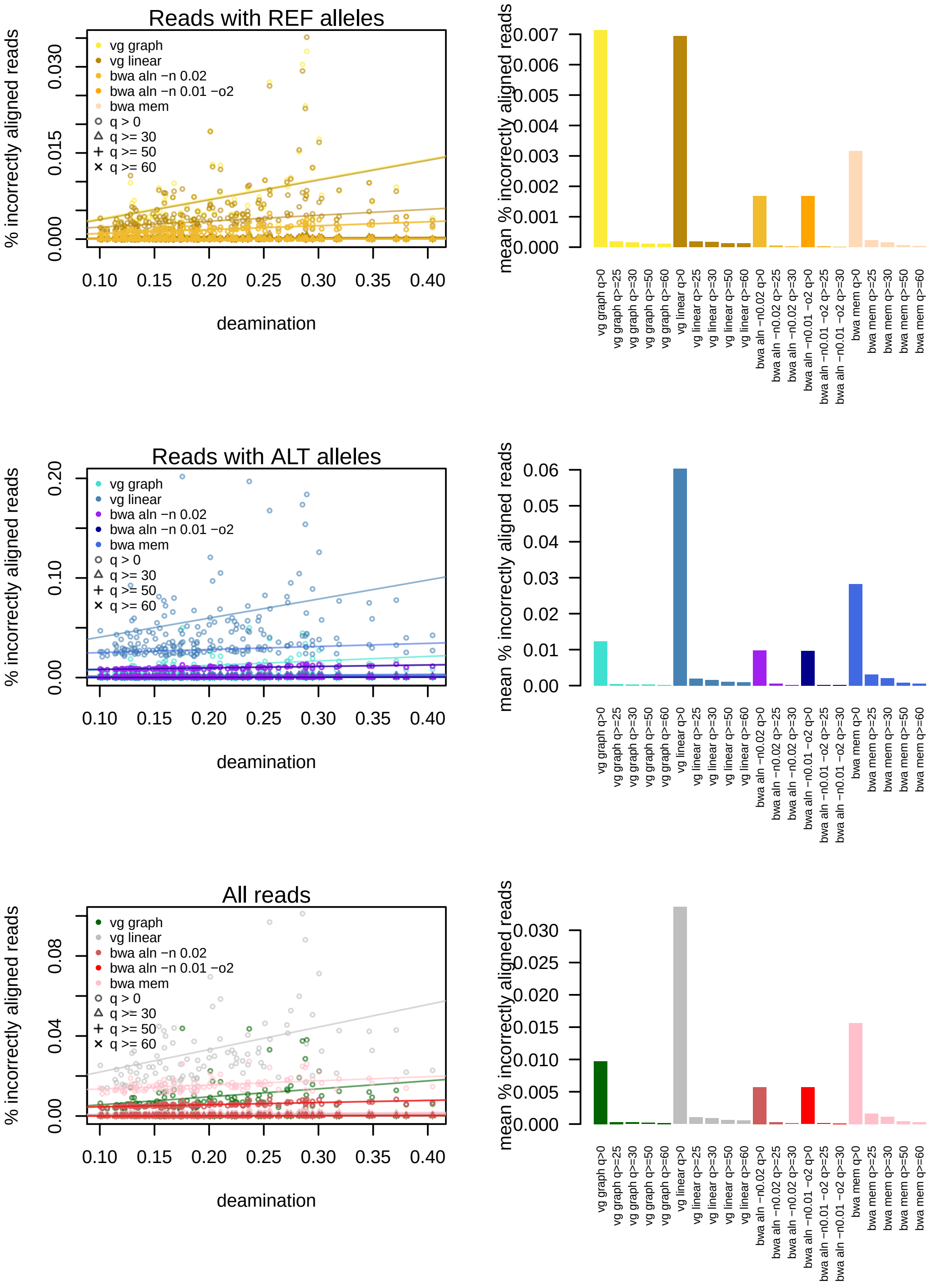

Fig. S6: Comparison of the percentage of incorrectly mapped reads between bwa (aln and mem), vg and vg linear reference using different parameters and filtered with different mapping quality thresholds (q>0, q≥30, q≥50 and q≥60). The plots on the left show the percentage of incorrectly mapped reads at increasing deamination rates, and the barplots on the right indicate the mean for each aligner and imposed mapping quality threshold.

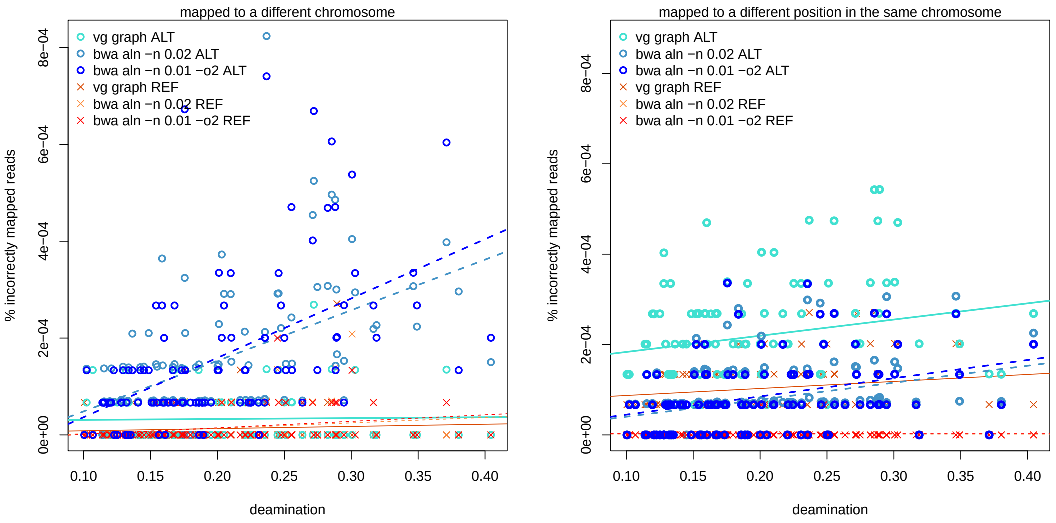

Fig. S7: Relationship between deamination and alignment error in vg (q≥50) and bwa aln (q≥30) alignments. Reads were simulated from chr11. The left side plot shows the percentage of reads incorrectly mapped to a different chromosome, and the one on the right shows the percentage of reads incorrectly mapped to a different position of the same chromosome they were simulated from.

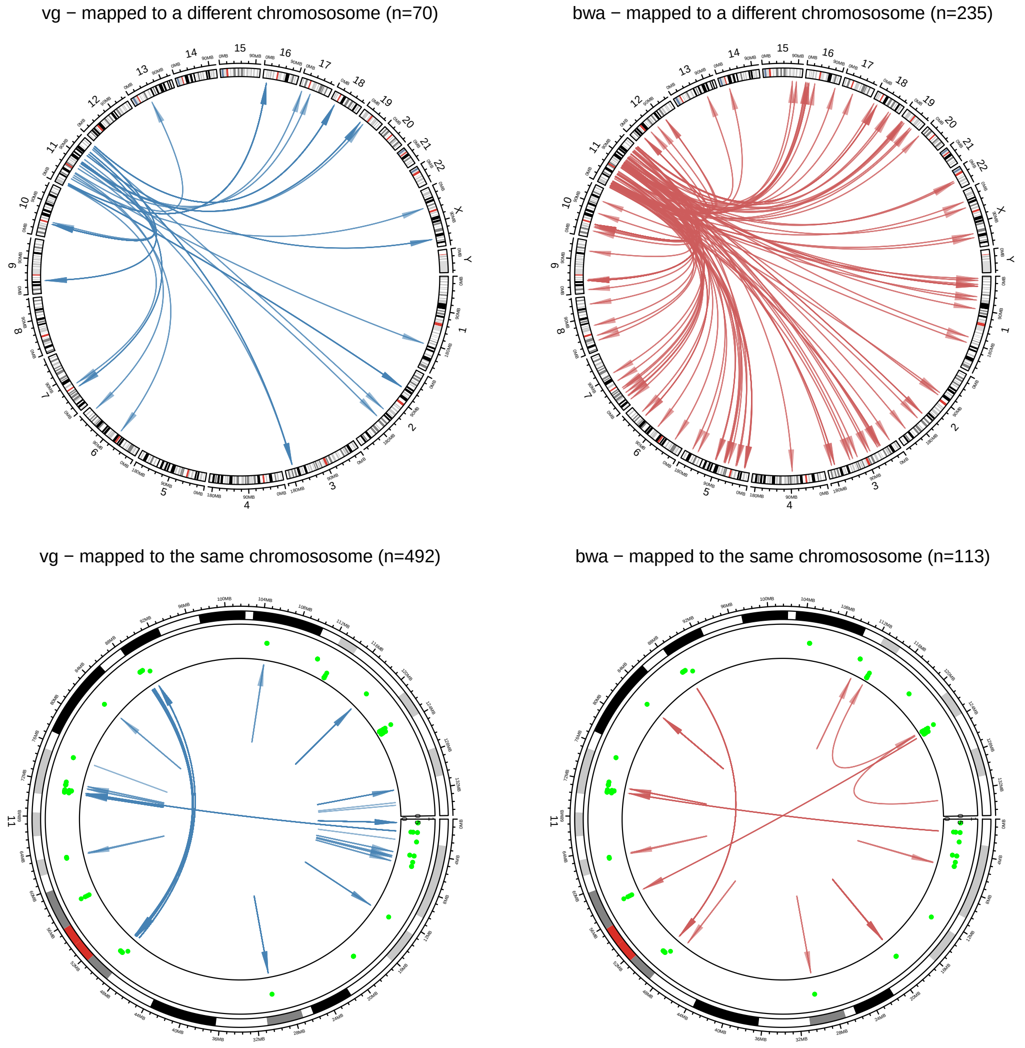

Fig. S8: Circos plots comparing the types of errors observed in all the simulated reads aligned with bwa aln (-n 0.02 q≥30) and vg graph (q≥50). Alignment errors are represented by arrows, with each arrow connecting the region where reads were simulated from and the regions where they were aligned to. The top circos plots show cases where reads were mapped to different chromosomes, while the bottom plots show cases where a read was aligned to a different coordinate within chr11. The vast majority of mapping errors occur in reads overlapping regions with low mappability. Green points indicate overlaps with low mappability (≤ 0.5) regions in the Mappability 50 track, downloaded from the UCSC table browser. A mappability of 0.5 means that a given read can be mapped equally well to two different places in the human reference genome. The numbers above each plot are the total error counts across all simulated individuals.

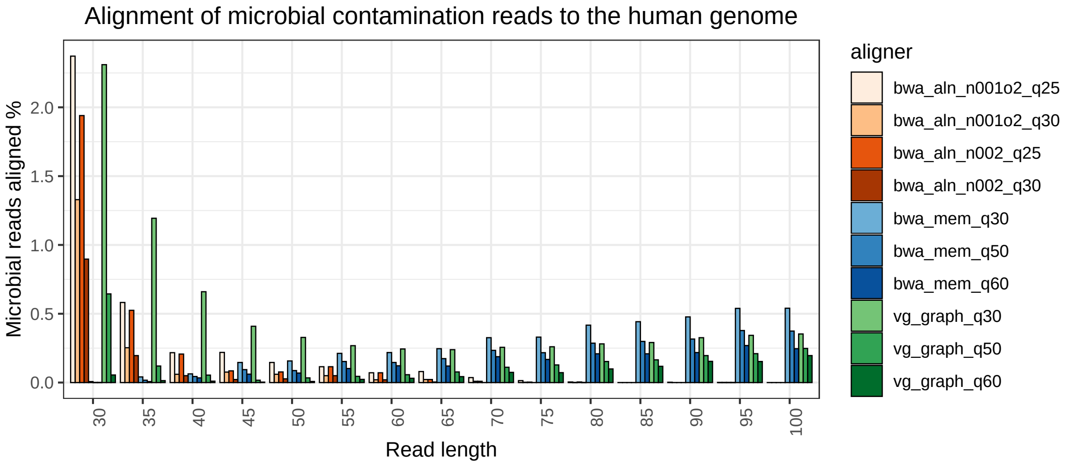

Fig. S9: Comparison of spurious alignment of simulated microbial sequences of different read lengths (30-100bp) between bwa aln and bwa mem to the human reference genome and vg to the 1000GP graph.

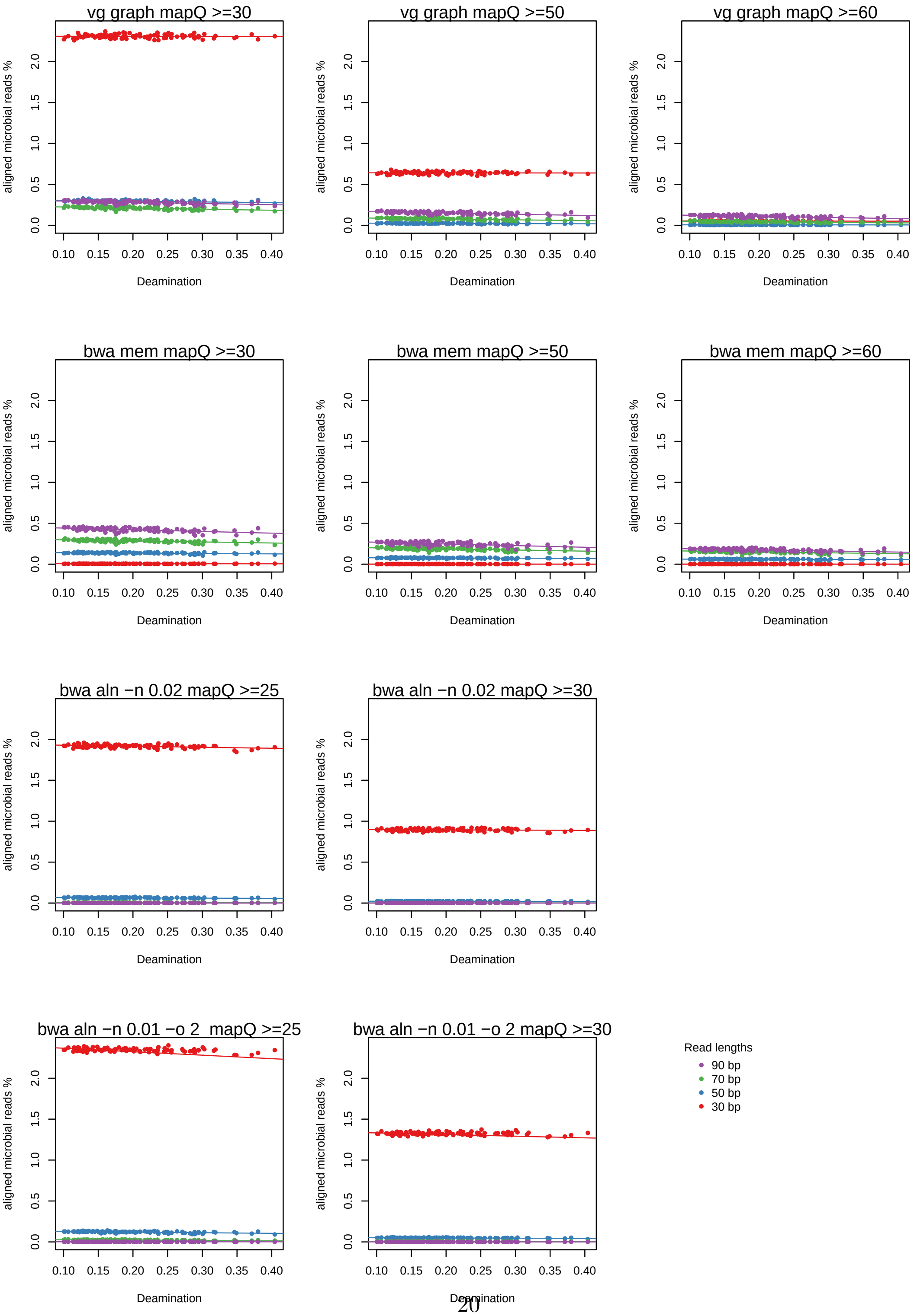

Fig. S10: Investigation of the effect of deamination levels in the spurious alignment of simulated microbial sequences of different read lengths (30, 50, 70 and 90bp) in bwa aln, bwa mem and vg.

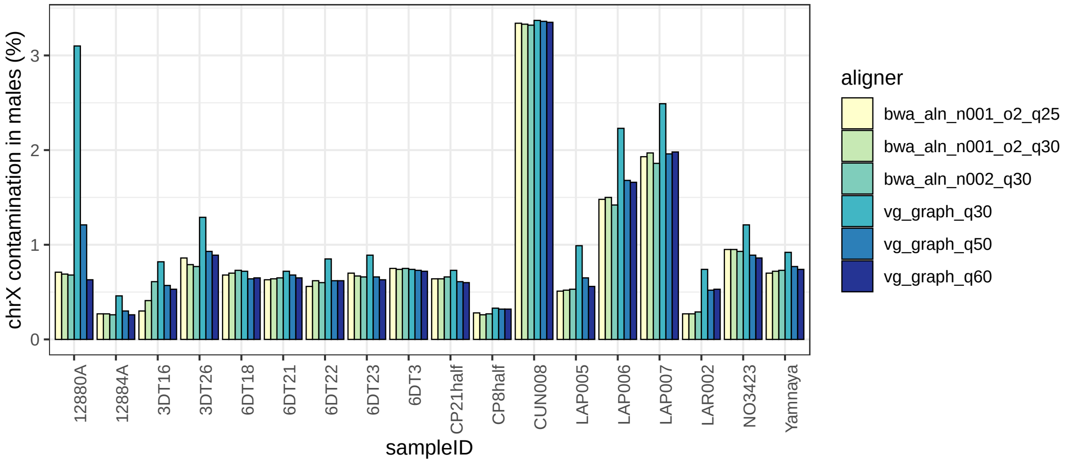

Fig. S11: Comparison of the fraction of X-chromosome contamination estimates in male samples aligned with vg graph and bwa aln -0.02 and -n 0.01 -o2 and filtered using different mapping quality thresholds.

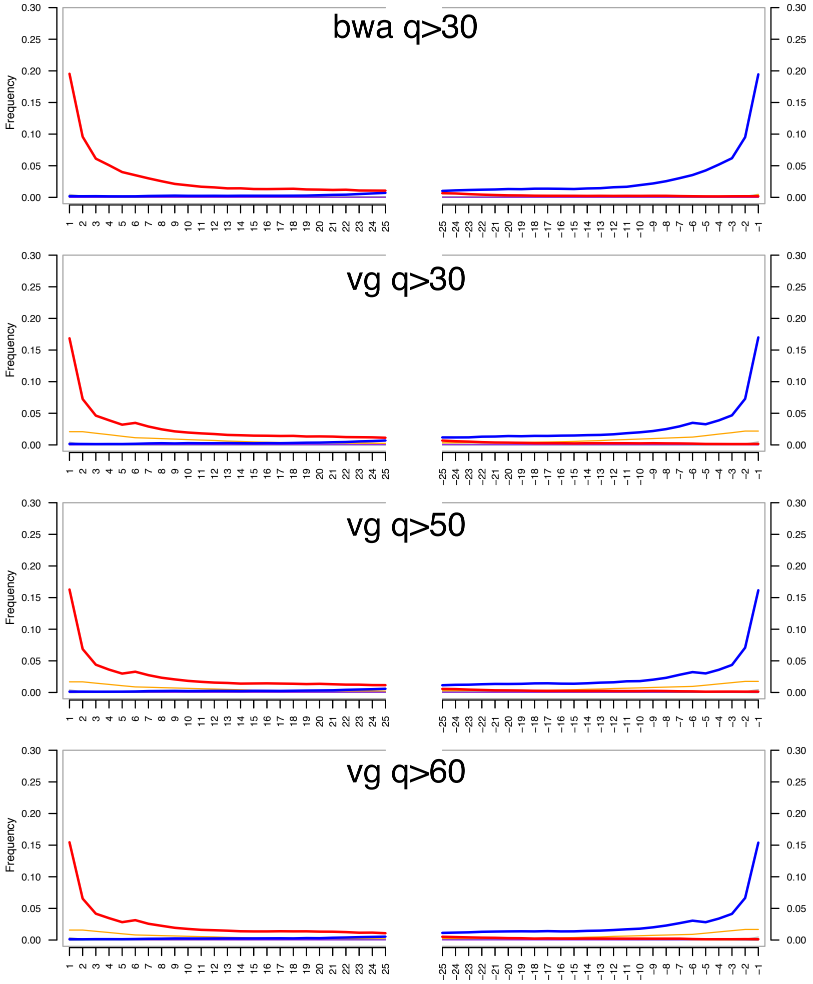

Fig. S12: Comparison between deamination patterns in sequence read data belonging to a high-coverage Yamnaya sample processed with vg and bwa aln (-n 0.02) and filtered with different mapping quality thresholds. Red: C to T substitutions; blue: G to A substitutions, and orange: soft-clipped bases.

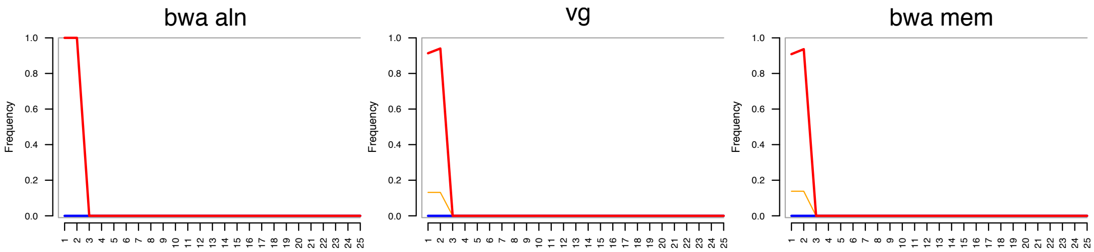

Fig. S13: Comparison of deamination and softclipping patterns in sequences with artificially introduced C to T changes in the 1st and 2nd nucleotides of the 5'-end of sequencing reads. Softclipping (orange curve) is more frequent in vg and bwa mem, leading to a slight reduction in the red curve which indicates C to T changes.

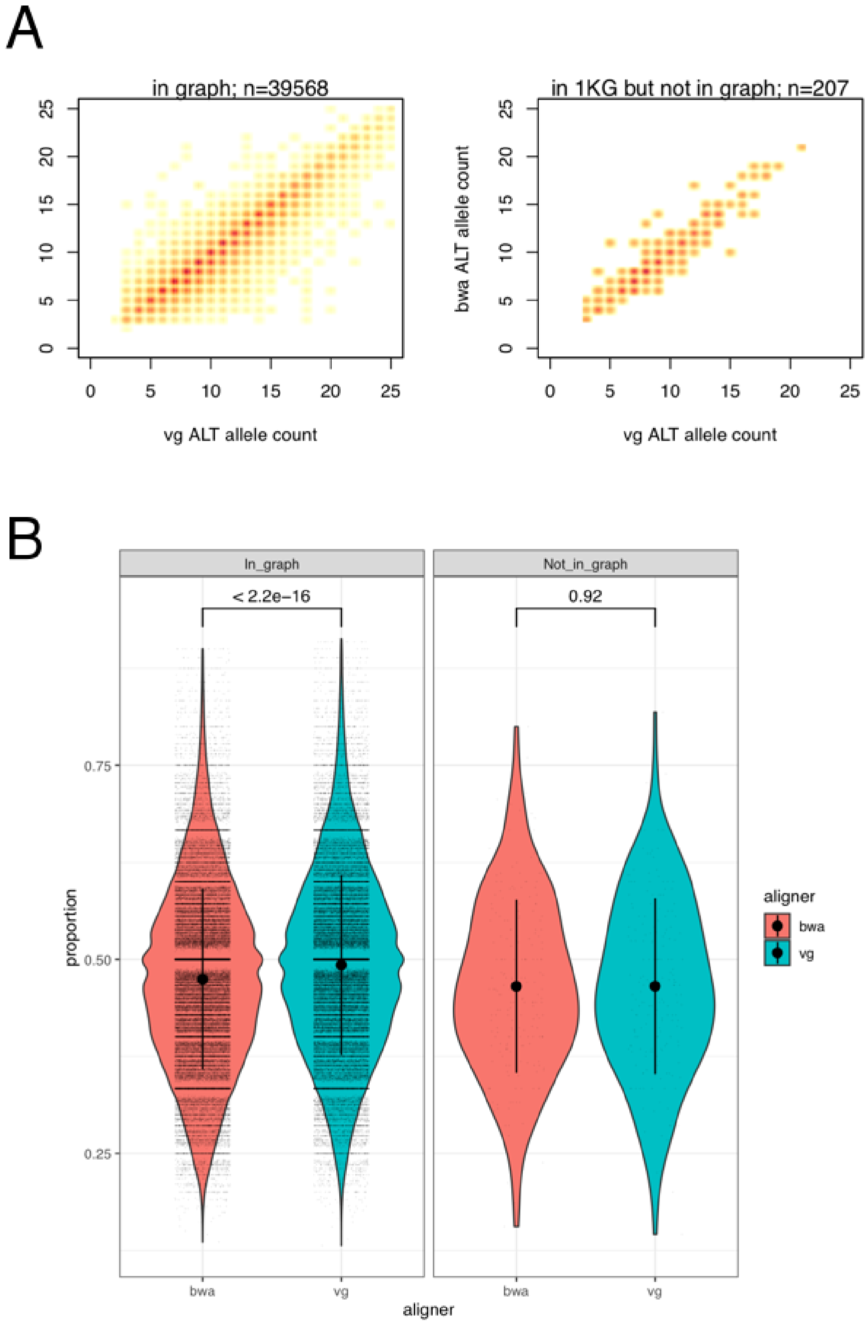

Fig. S14: Comparison of alternate allele support in heterozygous transversions of chr1 called from the Yamnaya sample aligned with `vg map` and `bwa aln -n 0.02` . A) Alternate allele counts in `bwa aln` and `vg map` aligned samples at SNPs present (left) or absent (right) in the graph. B) Alternate allele proportion in `vg map` and `bwa aln` aligned bams at SNP sites present (left) or absent (right) in the graph.

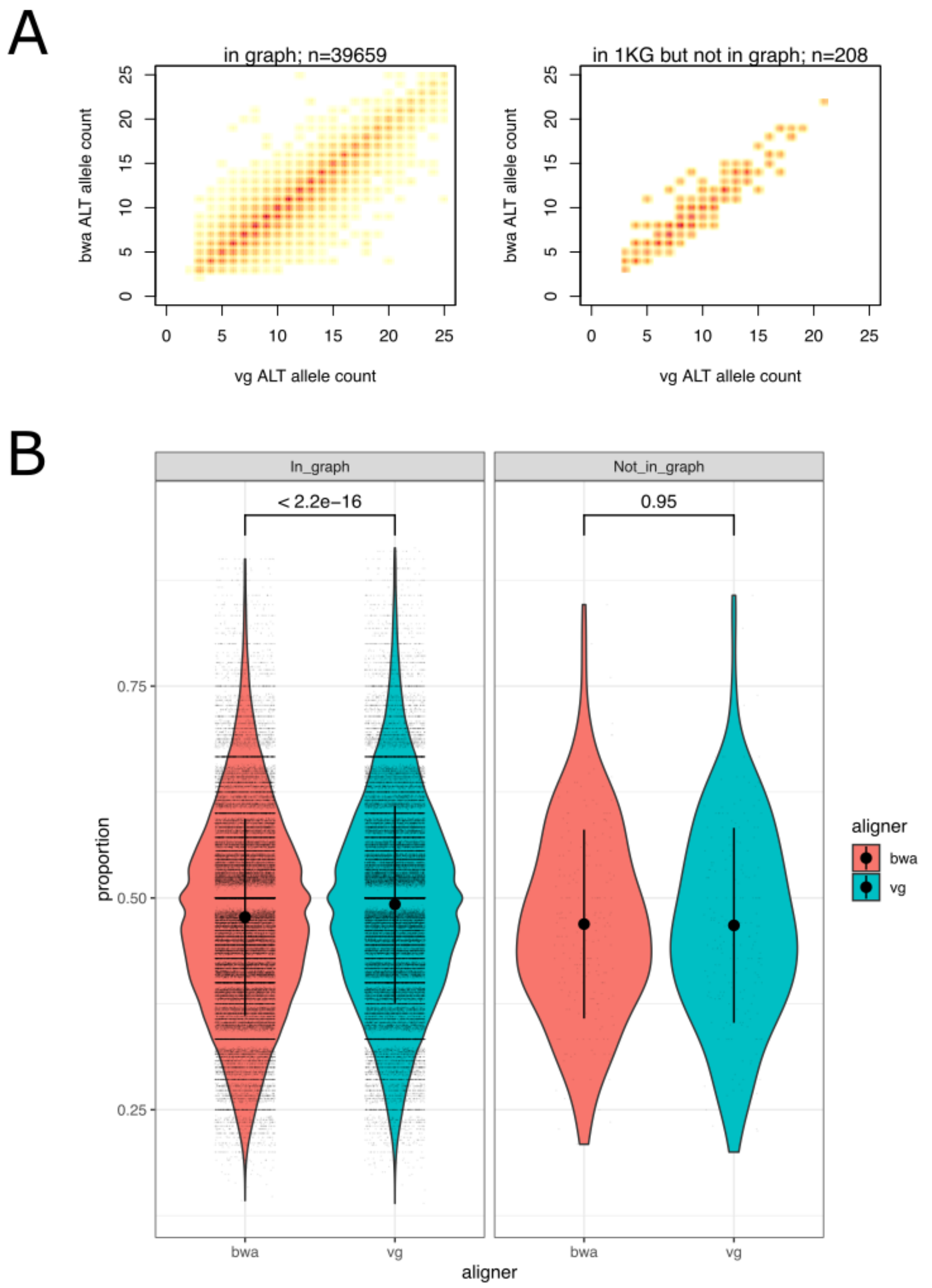

Fig. S15: Comparison of alternate allele support in heterozygous transversions of chr1 called from the Yamnaya sample aligned with `vg map` and `bwa aln -n 0.01 -o2` . A) Alternate allele counts in `bwa aln` and `vg map` aligned samples at SNPs present (left) or absent (right) in the graph. B) Alternate allele proportion in `vg map` and `bwa aln` aligned bams at SNP sites present (left) or absent (right) in the graph.

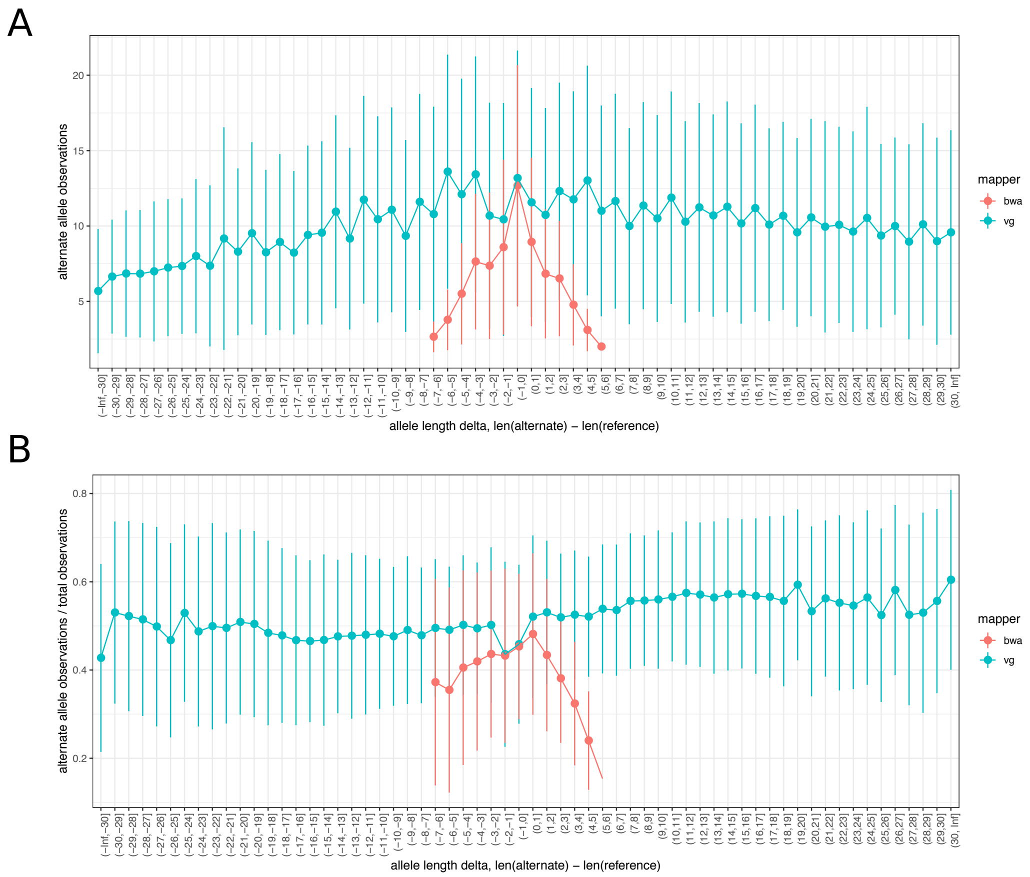

Fig. S16: Comparison of indel calling between the Yamnaya sample processed with vg graph and bwa aln (-n 0.02) to demonstrate the relationship between allele length and reference bias. While with vg it is possible to call alternate alleles at indels of various lengths, bwa shows a strong reference bias to insertions and deletions greater than a few bp. A) Alternate allele observations at indels. B) Fraction of alternate alleles in all indel observations. Alleles were binned by their relative length to the reference allele.

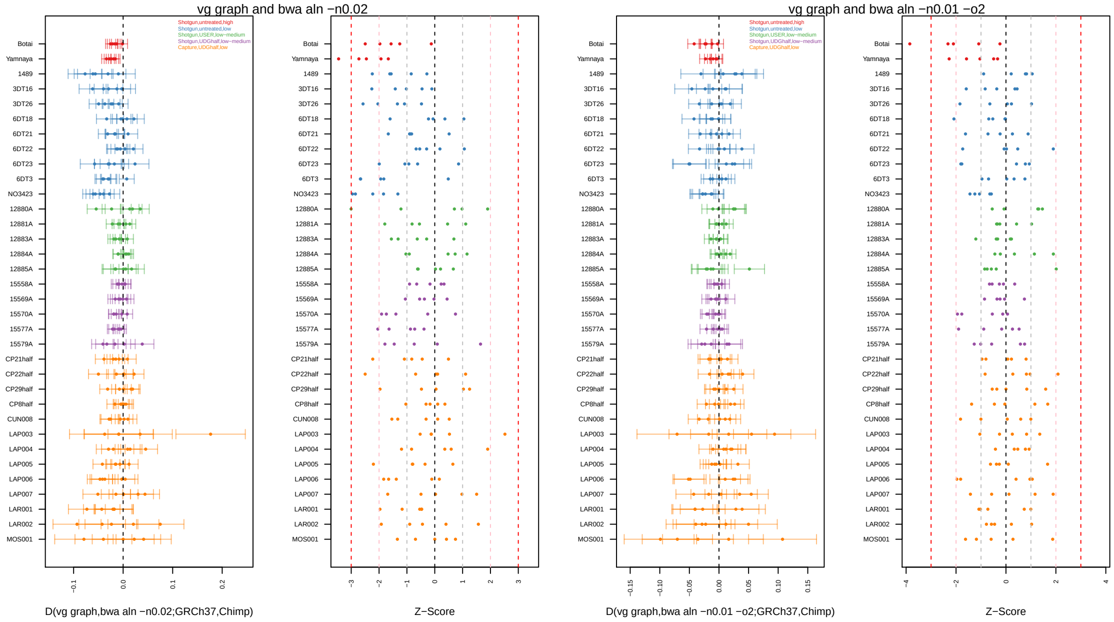

Fig. S17: D-statistic test of reference bias comparing vg and bwa, using Chimp as an outgroup and including transversion SNPs only. The left two panels compare vg graph (mapQ50) and bwa aln -n0.02 (mapQ30) in terms of bias towards the reference and the right two panels compare vg graph (mapQ50) and bwa aln -n0.01 -o2 (mapQ25).

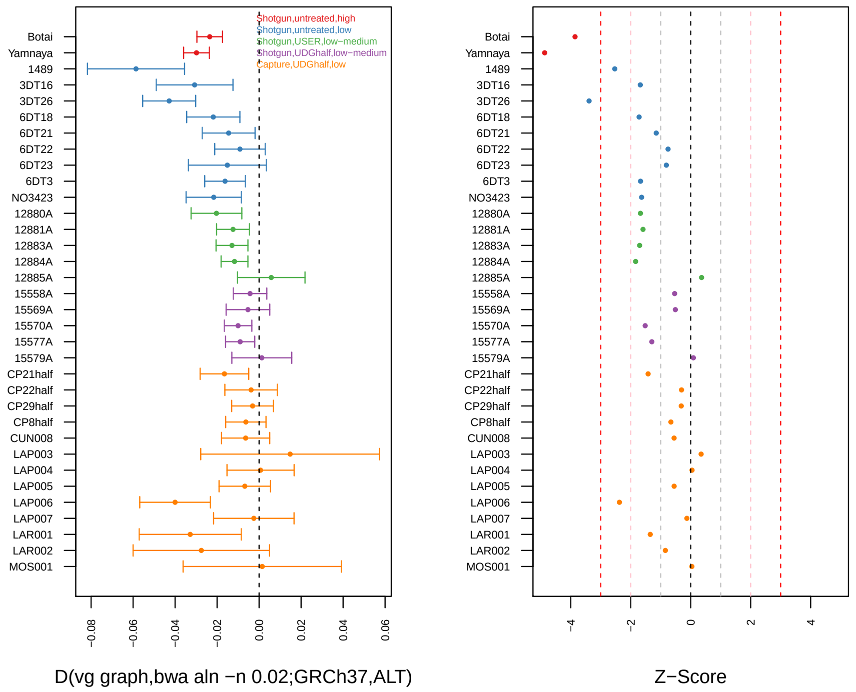

Fig. S18: D-statistics of the form D(vg graph, bwa aln -n 0.02; GRCh37,alternate allele) estimated for 34 ancient individuals, using transversion SNPs only. The figure label indicates the type of sequencing, enzymatic treatment and genomic coverage for each sample.

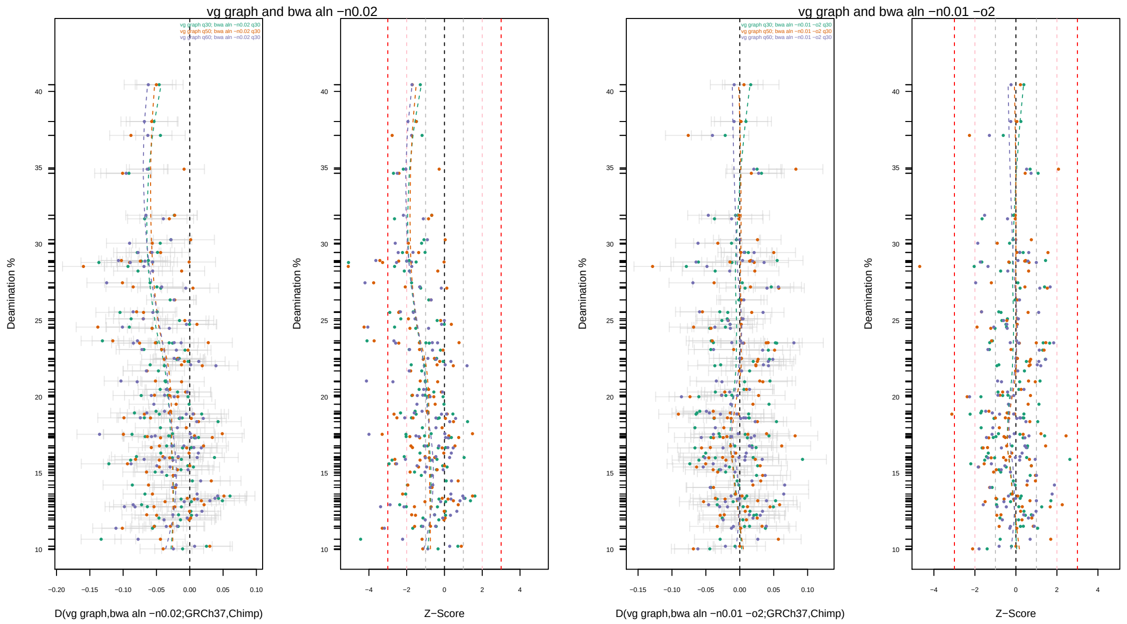

Fig. S19: *D*-statistic based ABBA-BABA test of reference bias based on simulated data aligned with vg graph and bwa aln, and using Chimp as an outgroup and including transversion SNPs only. The left two panels compare vg graph (mapQ50) and bwa aln -n0.02 (mapQ30) in terms of bias towards the reference and the right two panels compare vg graph (mapQ50) and bwa aln -n0.01 -o2 (mapQ25).

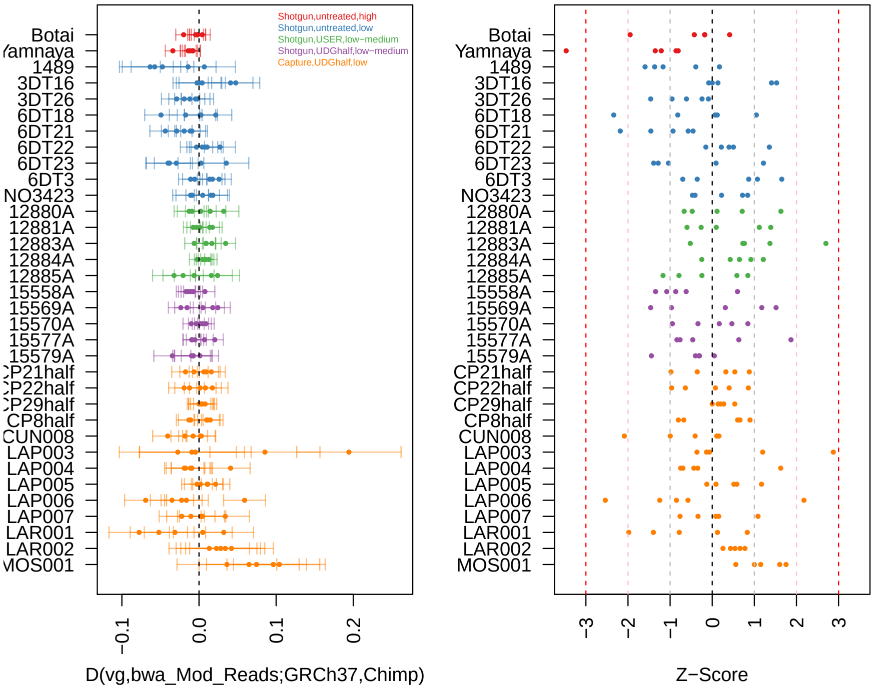

Fig. S20: D-statistic based ABBA-BABA test of reference bias comparing bwa-modread and vg using Chimp as an outgroup and estimated with transversion SNPs only. We included 5 replicates per ancient sample to account for the randomness in pseudo-haploid genotype generation.

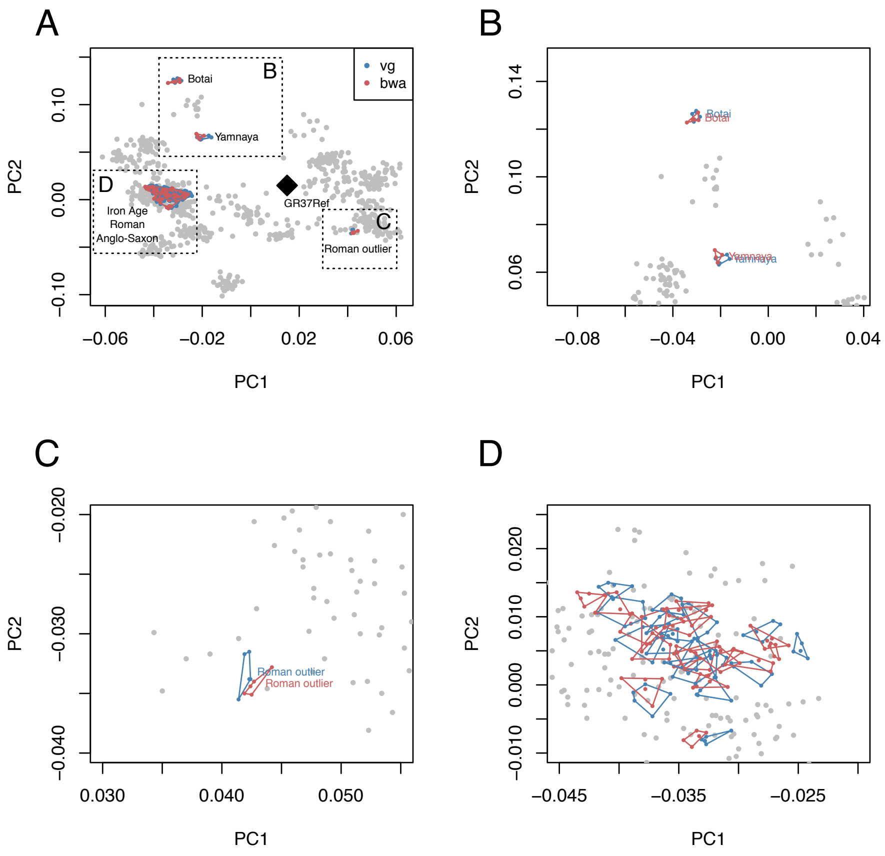

Fig. S21: Principal Component Analysis estimated with present-day West Eurasians from the Human Origins dataset with ancient samples aligned with either vg graph (blue) or bwa aln (-n 0.02) (red) projected onto it. 5 replicates of random allele sampling for each one of the ancient samples were included in the analysis. A) Full view of the PCA. Zoomed in PCA regions: B) Botai and Yamnaya, C) Roman outlier, D) Iron Age, Roman and Anglo-Saxon samples.

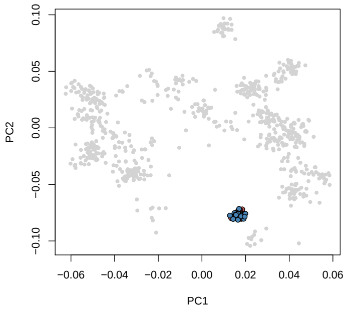

(a) Full PCA.

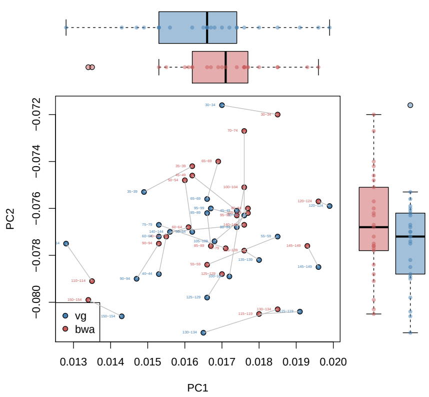

(b) PCA zoomed into the coordinates of the Yamnaya sample.

Fig. S22: PCA estimated with West Eurasian populations from the Human Origins dataset and with the projected Yamnaya sample, aligned with either vg graph and bwa aln (-n 0.02), and divided into 25 different read length bins (30-155bp). Present-day populations are coloured in grey. Yamnaya pseudo-haploid genotypes obtained from vg (blue) or bwa aln (red) aligned reads. Each 5 bp bin is represented by a point. Genotypes called from the same read length bin in bwa aln and vg are connected by a grey line.

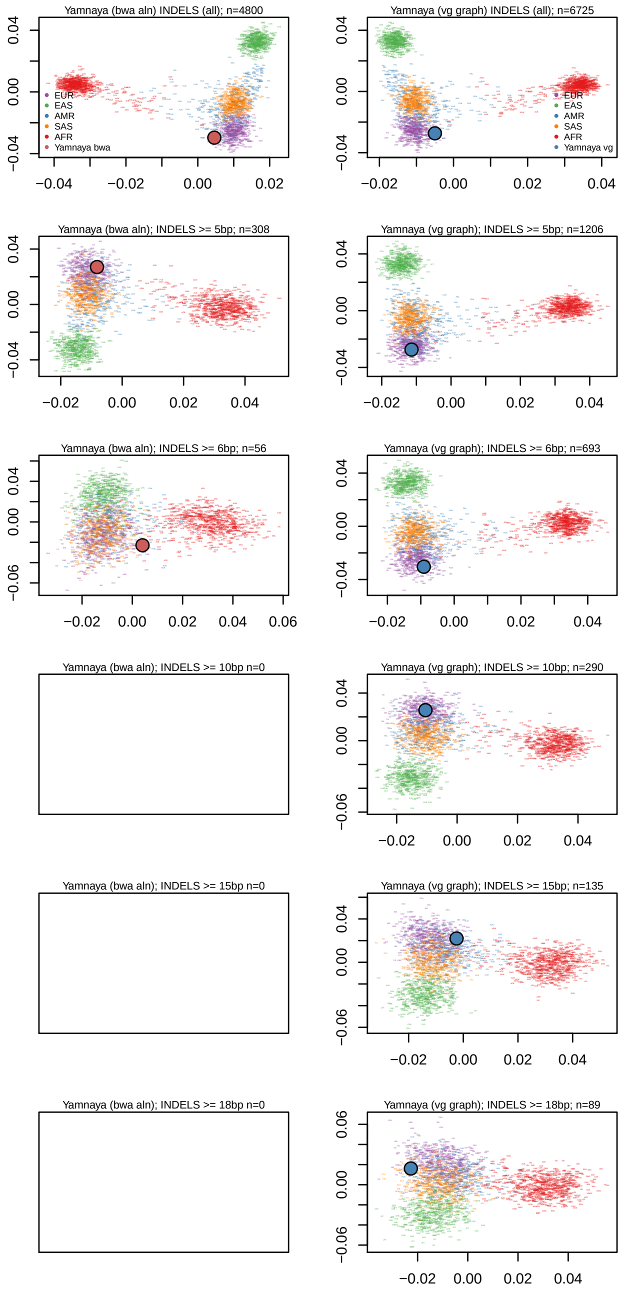

Fig. S23: Principal Component Analysis estimated with chr21 indels from the 1000 Genomes project. The indels used for analysis were restricted to those identified either in Yamnaya aligned with bwa aln (-n 0.02,left) or with vg graph. While PCAs estimated with vg indels maintain a clear clustering of the 1000 Genomes superpopulations across a wide range of indel allele lengths, with bwa this is not the case, given it does not recover longer indels than a few basepairs. The x and y axes are PC1 and PC2. Some plots were intentionally left blank to demonstrate the inability of bwa to call indels greater than 6 basepairs.
